# Exploratory examination of inflammation state, immune response and blood cell composition in a human obese cohort to identify potential markers predicting cancer risk

**DOI:** 10.1371/journal.pone.0228633

**Published:** 2020-02-06

**Authors:** Ingrid Elisia, Vivian Lam, Brandon Cho, Mariah Hay, Michael Yu Li, Jordanna Kapeluto, Tom Elliott, David Harris, Luke Bu, William Jia, Hilary Leung, William Mohn, Gerald Krystal

**Affiliations:** 1 The Terry Fox Laboratory, British Columbia Cancer Agency, Vancouver, British Columbia, Canada; 2 Department of Medicine, Division of Endocrinology, University of British Columbia, Vancouver, British Columbia, Canada; 3 B.C Diabetes, Vancouver, British Columbia, Canada; 4 Richmond General Hospital Metabolic and Bariatric Surgery, Richmond, British Columbia, Canada; 5 Brain Research Centre, University of British Columbia, Vancouver, British Columbia, Canada; 6 Microbiome Insights, Vancouver, British Columbia, Canada; 7 Department of Microbiology & Immunology, University of British Columbia, Vancouver, British Columbia, Canada; Universidad de Malaga, SPAIN

## Abstract

Obesity has reached epidemic proportions and is often accompanied by elevated levels of pro-inflammatory cytokines that promote many chronic diseases, including cancer. However, not all obese people develop these diseases and it would be very helpful to identify those at high risk early on so that preventative measures can be instituted. We performed an extensive evaluation of the effects of obesity on inflammatory markers, on innate and adaptive immune responses, and on blood cell composition to identify markers that might be useful in distinguishing those at elevated risk of cancer. Plasma samples from 42 volunteers with a BMI>35 had significantly higher CRP, PGE_2_, IL-1RA, IL-6 and IL-17 levels than 34 volunteers with normal BMIs. Of the cytokines and chemokines tested, only IL-17 was significantly higher in men with a BMI>35 than women with a BMI>35. As well, only IL-17 was significantly higher in those with a BMI>35 that had type 2 diabetes versus those without type 2 diabetes. Whole blood samples from participants with a BMI>35, when challenged with *E*. *coli*, produced significantly higher levels of IL-1RA while HSV-1 challenge resulted in significantly elevated IL-1RA and VEGF, and a non-significant increase in G-CSF and IL-8 levels. T cell activation of PBMCs, via anti-CD3 plus anti-CD28, resulted in significantly higher IFNγ production from volunteers with a BMI>35. In terms of blood cells, red blood cell distribution width (RDW), monocytes, granulocytes, CD4^+^T cells and Tregs were all significantly higher while, natural killer (NK) and CD8^+^ T cells were all significantly lower in the BMI>35 cohort, suggesting that obesity may reduce the ability to kill nascent tumor cells. Importantly, however, there was considerable person-to-person variation amongst participants with a BMI>35, with some volunteers showing markedly different values from controls and others showing normal levels of many parameters measured. These person-to-person variations may prove useful in identifying those at high risk of developing cancer.

## Introduction

Obesity has reached epidemic proportions worldwide, increasing the risk of many chronic diseases, including type 2 diabetes, rheumatoid arthritis, dementia, cardiovascular disease and cancer [1]. As well, obesity has been shown to increase the risk of infections and to negatively impact the outcome of infections once they are established [2], suggesting a dysregulation of the immune system. This is consistent with obesity being associated with low-grade chronic inflammation (CI) and an impaired immune response to both bacterial and viral infections [3] and to vaccinations [4]. The increase in baseline inflammation is also thought to predispose people with obesity to an increased risk of cancer [5]. However, not all obese people develop cancer and up to 30% of obese people have been shown to be metabolically healthy (i.e., have high insulin sensitivity, low abdominal adiposity and low levels of CI [6]. Thus, it would be very helpful to identify those at highest risk for cancer and other chronic diseases as early as possible so that preventative measures can be instituted.

With this in mind, we recently optimized assays to measure an individual’s level of CI and immune function and investigated how these change with age in healthy volunteers with normal body mass indexes (BMIs) (within the 18.5–24.9 range) [7]. In the current study, we used these assays to compare participants with BMIs>35 to normal BMI controls. Specifically, we compared the basal inflammatory state of participants by quantifying their plasma levels of cytokines/chemokines and characterizing their immune cells in peripheral blood. We also compared their innate immune response by challenging their blood, *ex-vivo*, with intact bacteria (*Escherichia coli*) and virus (herpes simplex virus-1 (HSV-1)) to simulate bacterial and viral infections, respectively. Lastly, we compared their adaptive T cell immune response by stimulating peripheral blood mononuclear cells (PBMCs) with anti-CD3 plus anti-CD28 and measuring the production of interferon γ (IFN-γ). These *ex vivo* challenges were carried out to further explore the concept that elevated basal levels of pro-inflammatory cytokines/chemokines impair immune responses to infections [4].

While it is known that obesity is associated with CI and a dysregulated innate and adaptive immune response, there is not as yet any simple immune markers that can distinguish people with BMIs>35 who have an increased risk of cancer from those who do not. However, in terms of basal levels, apart from IL-1RA, TGFβ and IL-10, which are anti-inflammatory, there is a general consensus that elevated cytokines/chemokines likely increase the risk of cancer [8]. Moreover, in terms of cytokine response to *E*. *coli* and HSV-1, a robust pro-inflammatory response likely suggests better clearance of infection. However, an overly robust response might indicate an increased susceptibility to autoimmune diseases and cancer. As well, since some studies have suggested that obesity leads to an immunosenescent profile similar to that seen in normal aging [9, 10] we set out in the current study to (a) compare levels of CI, immune function and blood cell components in participants with BMIs>35 to people with normal BMIs and to (b) compare results obtained from this cohort to previously published data from our normal aging study [7]. The ultimate aim of these studies is to identify novel markers that are characteristic of people with a BMI>35 and determine if they can be used, in the future, to predict cancer risk. The results of these studies are presented herein.

## Materials and methods

### Human participants and blood collection

All studies were approved by the joint Clinical Research Ethics Board of the University of British Columbia and BC Cancer (#H12-00727). Forty two volunteers with BMIs>35 (class 2 obesity) and 34 healthy, non-smoking volunteers with BMIs between 18.5–24.9 were recruited. We chose volunteers with a BMI>35 (class 2 obesity or severely obese) to reduce the probability of recruiting a lean volunteer with high muscle mass, such as that observed in athletes. All participants provided informed written consent. Participants were asked to refrain from consuming non-steroidal anti-inflammatory drugs for 2 days prior to their blood draw and were excluded from the study if they had recent infections or traumatic injuries. Blood samples were collected between 8:30 am and 10:00 am to avoid reported changes in cytokine secretion with diurnal rhythms [11]. Blood was drawn into 6 mL K_2_EDTA Vacutainer tubes (cat. no. 367861, BD, Mississauga, ON) and endotoxin-free [12] glass sodium heparin Vacutainer tube (cat. no. 366480, BD, Mississauga, ON).

### Human blood assay

Human blood samples collected in EDTA or sodium heparin containing glass tubes were mixed gently, kept at 23°C and aliquoted within 2 h of collection into 96-well round bottom tissue culture plates. 50 μL of blood was added to individual wells along with 10 μL of either PBS (Control), *Escherichia coli* (*E*. *coli*, One Shot INV 110, Life Technologies, Burlington, ON) at a final concentration of 2 x 10^4^ cells/mL, or HSV-1 G207 at a multiplicity of infection (MOI) of 0.06 (relative to total white blood cell numbers). Plates were then incubated for 7 h in a 5% oxygen, humidified incubator at 37ºC. Following incubation, 100 μL of PBS was added to each well, the cells were then thoroughly resuspended and centrifuged at 424 x *g* at 4ºC for 5 min. Supernatants were collected and immediately frozen at -80ºC.

### Luminex analysis

A custom magnetic Luminex assay panel from Life Technologies was used to assess the levels of the following 15 cytokines and chemokines in human plasma: Interleukin (IL)-1β, Granulocyte-colony stimulating factor (G-CSF), IL-10, IL-13, IL-6, IL-17, macrophage inflammatory protein (MIP)1α, Vascular endothelial growth factor (VEGF), interferon (IFN)γ, IL-12p70, IFNα, IL-1RA, tumor necrosis factor (TNF)α, IL-4 and IL-8. Frozen samples were thawed and centrifuged (1000 x g at 4ºC for 10 min) before testing. Plasma samples were incubated with antibody beads overnight at 4ºC. Assay plates were read according to the manufacturer’s instructions using a BioPlex 100 instrument utilizing Bio-Plex Manager 6.0 software (Bio-Rad Laboratories, Mississauga, ON).

### MesoScale analysis

Undiluted plasma samples were analyzed using a Mesoscale Discovery (MSD) V-PLEX Pro-inflammatory Panel 1 (human) kit for IL-6 quantification and Cytokine Panel 1 (human) kits for IL-17 and VEGF quantification (K15049D & K15050D, Mesoscale Discovery, Gaithersburg, MD) according to the manufacturer’s instructions and as previously described by Idborg et al [13]. Briefly, samples were incubated on the MSD plates for 2 h at 23°C with shaking. Plates were washed and incubated an additional 2 h with detection antibodies. After washing, 2× Read buffer T was added to each well and the plate analyzed in a QuickPlex SQ 120 model no. 1300. Calibrator and plasma samples were analyzed in duplicates. Using the MSD Workbench software the response of the calibrator concentrations was plotted as log signal unit on the vertical (Y) axis versus log concentration on the horizontal (X) axis.

### PGE_2_ and CRP measurements

ELISAs for prostaglandin E_2_ (PGE_2_) (Cat #514010, Cayman Chemical Company, Ann Arbor, MI), using EDTA-containing blood and C-reactive protein (CRP) (Cat # DCRP00, R&D Systems, Minneapolis, MN), using heparin-containing blood [7] were performed according to the manufacturers’ instructions.

### Blood differential counts

Blood differential cell counts were carried out on fresh whole blood collected in EDTA rather than heparin tubes to avoid heparin-induced aggregation of platelets [14], using a Coulter Ac•T diff2^TM^ Hematology Analyzer (Beckman-Coulter Corp., Miami, FL).

### Immunophenotyping

Human peripheral blood mononuclear cells (PBMCs) were isolated from heparinized whole blood by density gradient centrifugation with Lymphoprep (StemCell Technologies, Vancouver, BC). The PBMCs were stained with GhostDye Violet 450 viability dye (Tonbo Biosciences, San Diego, CA) for 30 min at 4ºC, washed once with PBS containing 2% FBS and 0.05% sodium azide (PFN), and blocked with anti-human CD32 Clone IV.3 (StemCell Technologies, Vancouver, BC) for 15 min at 23°C. This was followed by staining of cell surface markers for 30 min at 23°C. The cells were then washed twice and resuspended in PFN followed by flow cytometric analysis. To identify regulatory T cells, cells were fixed and permeabilized using the FoxP3 Staining Buffer Set (eBioscience, San Diego, CA). The cells were stained with the FoxP3 antibody overnight at 4ºC, washed once with PFN and analyzed by flow cytometry. All analysis was performed using a BD LSR Fortessa flow cytometer (BD Biosciences) and data analysis was performed using FlowJo software V10.2 (FlowJo, Ashland, OR). The antibodies used were: CD8-PE (clone SK1) and CD3-FITC (clone SK7) from StemCell Technologies, Vancouver, BC; CD45-FITC (Hle1), CD28-APC (clone CD28.2), CD4-PE-Cy7 (clone SK3), CD25-BB515 (clone 2A3), CD127-AF647 (clone HIL-7R-M21) and FoxP3-PE (clone 236A/E7) from BD Biosciences, Mississauga, ON; CD56-APC (clone CMSSB) from eBioscience, San Diego, CA.

### T cell activation

PBMCs isolated as above were counted using a Vi-Cell XR cell viability analyzer (Beckman Coulter, Brea, CA) and resuspended at 10^6^ cells/mL in RPMI + 10% autologous plasma + 100 U/mL penicillin/streptomycin. The PBMCs were aliquoted (50 μl/well) into 96-well flat-bottom tissue culture plates that were pre-coated overnight with 0.5 μg/mL of anti-human CD3 (clone OKT3, eBioscience, San Diego, CA). Anti-human CD28 (clone CD28.2, eBioscience, San Diego, CA) at a final concentration of 2 μg/mL was then added to each well, and the plates incubated in a humidified incubator at 5% CO_2_, 37ºC for 4 days. The plates were then centrifuged at 300 x g in a Beckman TJ-6 centrifuge for 5 min and the supernatants collected for IFNγ analysis by ELISA (BD Biosciences, San Diego, CA).

### Statistical analysis

**Fig 1:**
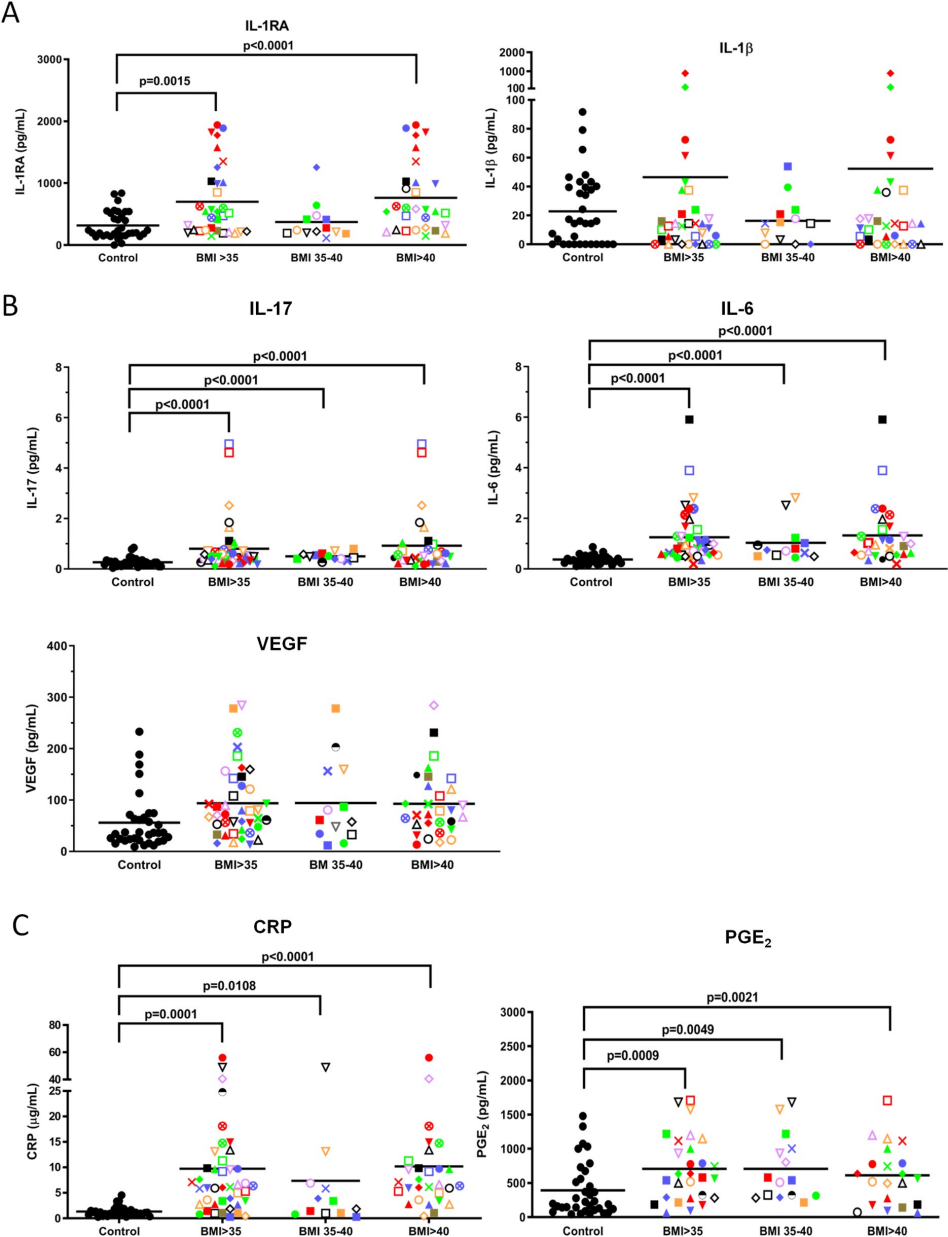
Plasma levels of IL-RA, IL-17, IL-6, CRP and PGE_2_ are higher in BMI>35 than control volunteers. (**A**) Blood samples were collected in heparin for all cytokines/chemokines except G-CSF (which was collected in EDTA) and subjected to Luminex analysis. ● = healthy controls; Each volunteer with a BMI>35 is identified with a unique symbol. All men are identified by squares. Results are expressed as the mean ± SEM. (**B**) MesoScale analyses of plasma samples from BMI>35 and control volunteers for IL-17, VEGF and IL-6 levels. (**C**) CRP levels in the left panel and PGE_2_ levels in the right.

**Fig 2:**
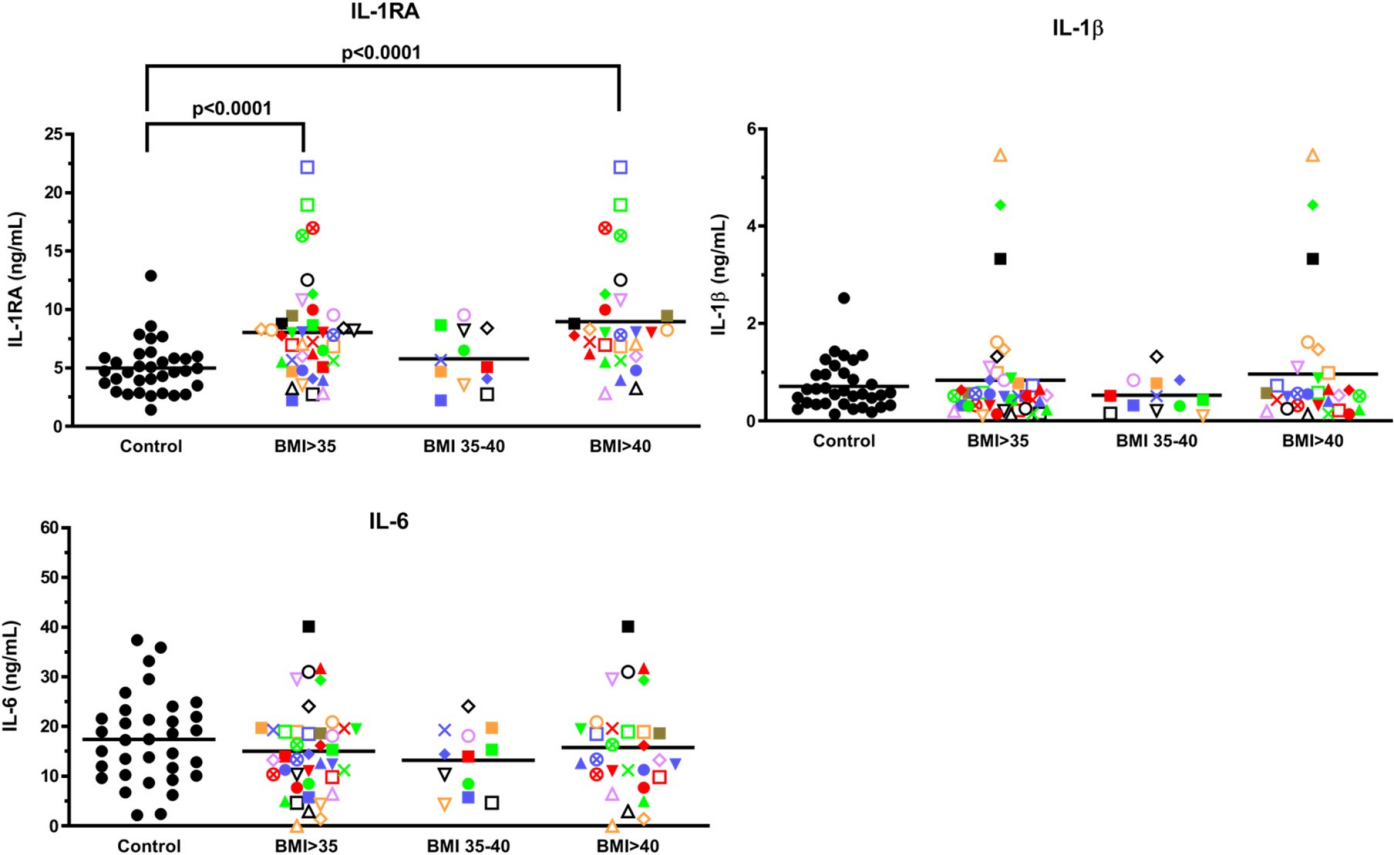
IL-1RA levels are elevated in whole blood samples from obese volunteers stimulated ex vivo with *E*. *coli*. **(A)** Whole fresh blood from 42 volunteers with BMIs>35 and 32 healthy people with BMIs between 18.5–24.9 was stimulated with *E*. *coli* for 7 h at 37°C and the conditioned plasma analyzed for cytokine/chemokine levels using Luminex beads. Each volunteer with a BMI>35 is identified with a unique symbol. Levels shown are for IL-1RA, IL-1β and IL-6. Results are expressed as the mean ± SEM.

**Fig 3:**
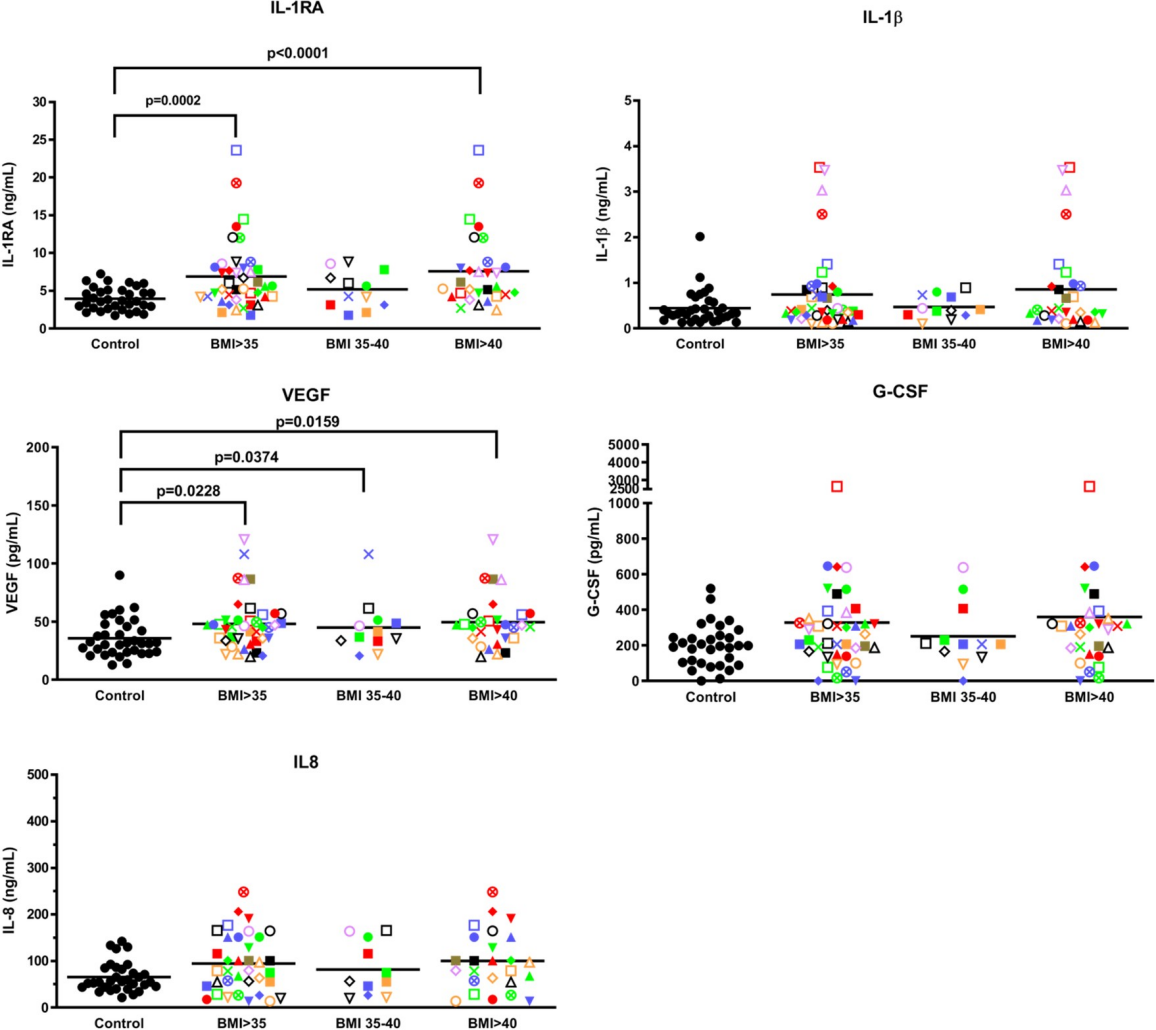
IL-1RA and VEGF are elevated in whole blood samples from BMI>35 volunteers stimulated *ex vivo* with HSV-1.

**Fig 4:**
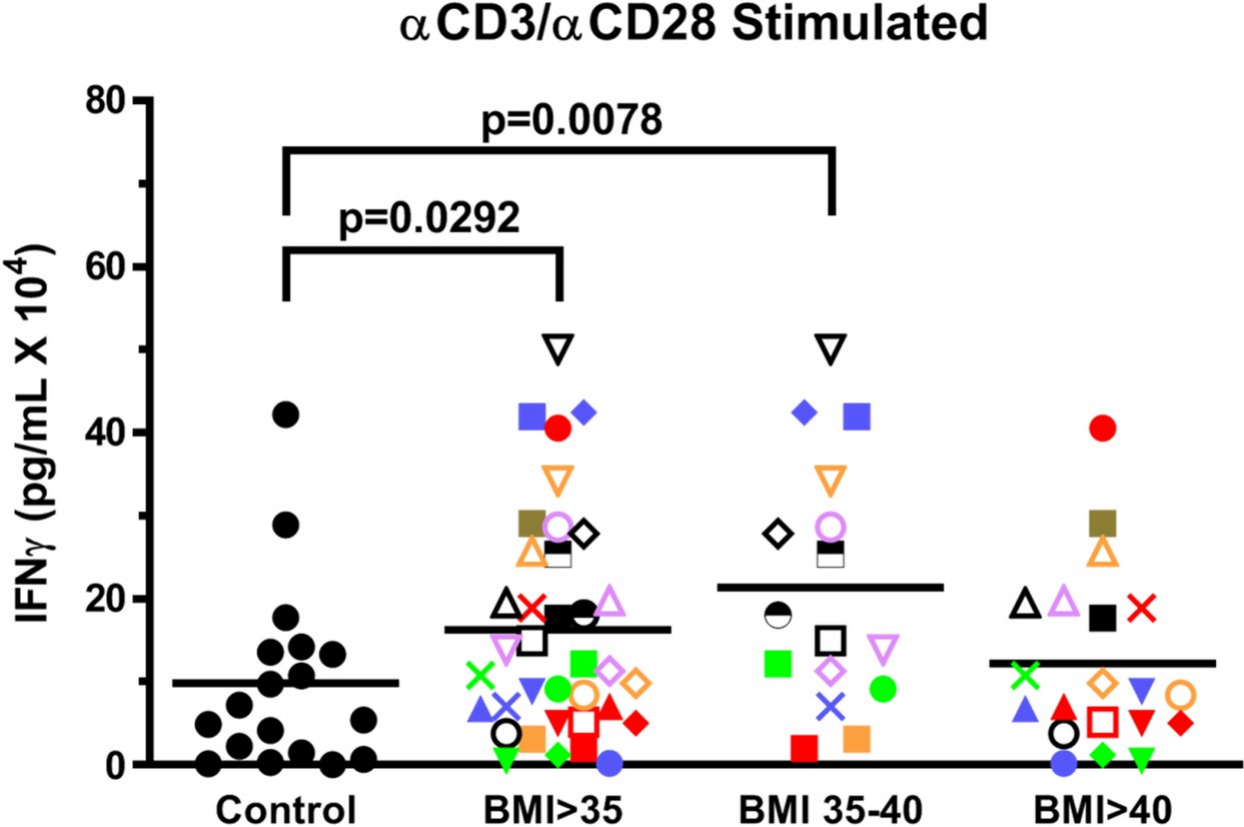
T cells from obese volunteers secrete more IFNγ than controls in response to anti-CD3 plus anti-CD28 stimulation. PBMCs from BMI>35 and control volunteers were stimulated with anti-CD3 plus anti-CD28 for 4 days in RPMI + 10% autologous plasma and the levels of IFNγ production determined. Each volunteer with a BMI>35 is identified with a unique symbol. Results are expressed as the mean ± SEM.

**Fig 5:**
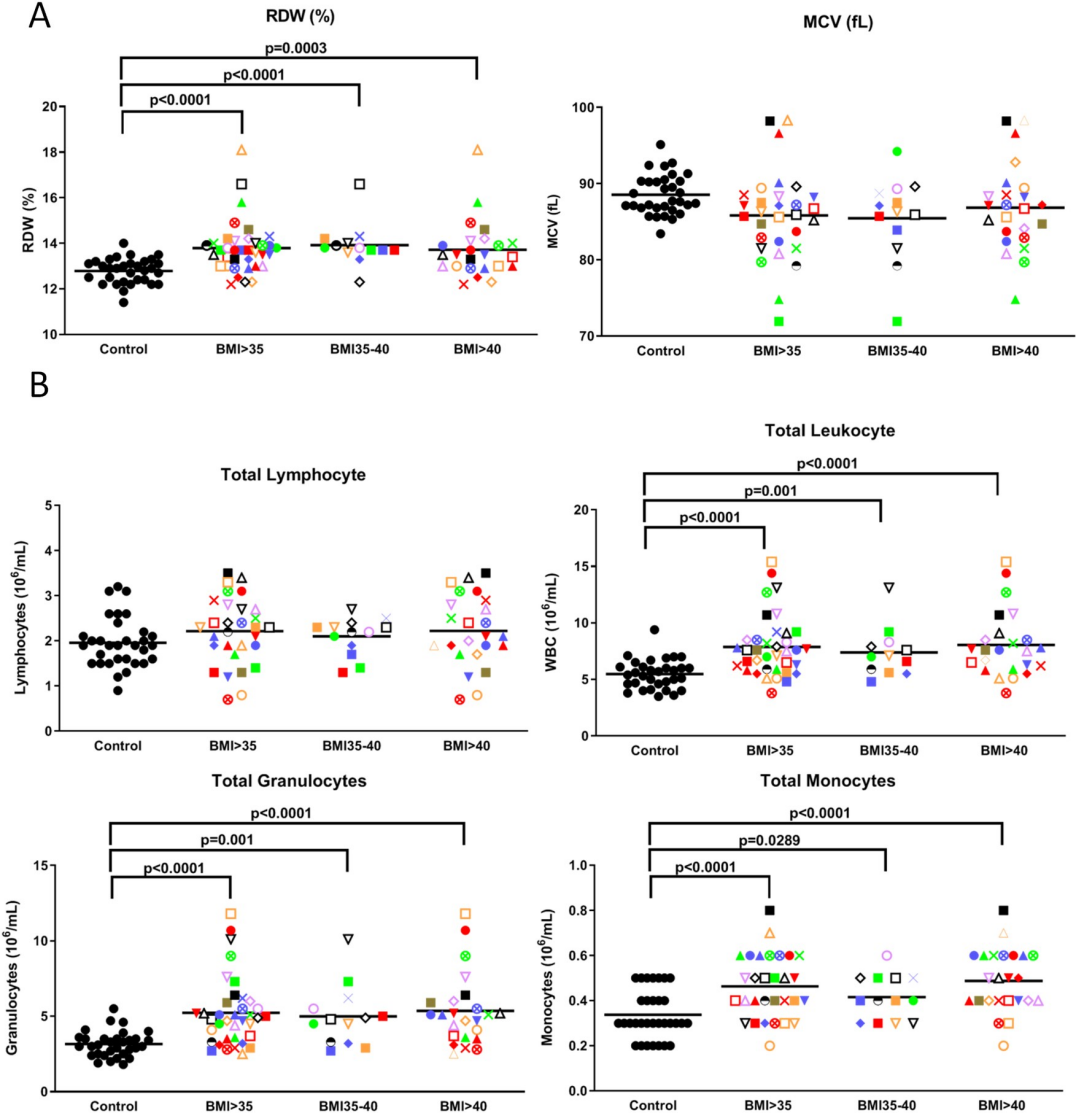
Obesity increases RDW, monocytes and granulocytes. Volunteers with BMIs>35, each with a unique identifying symbol, were compared with controls for **(A)** RDW and MCV, **(B)** total lymphocytes, leukocytes, granulocytes and monocytes. Each volunteer with a BMI>35 is identified with a unique symbol. Results are expressed as the mean ± SEM. P values of the correlation matrix are described in **S3 Table**.

**Fig 6:**
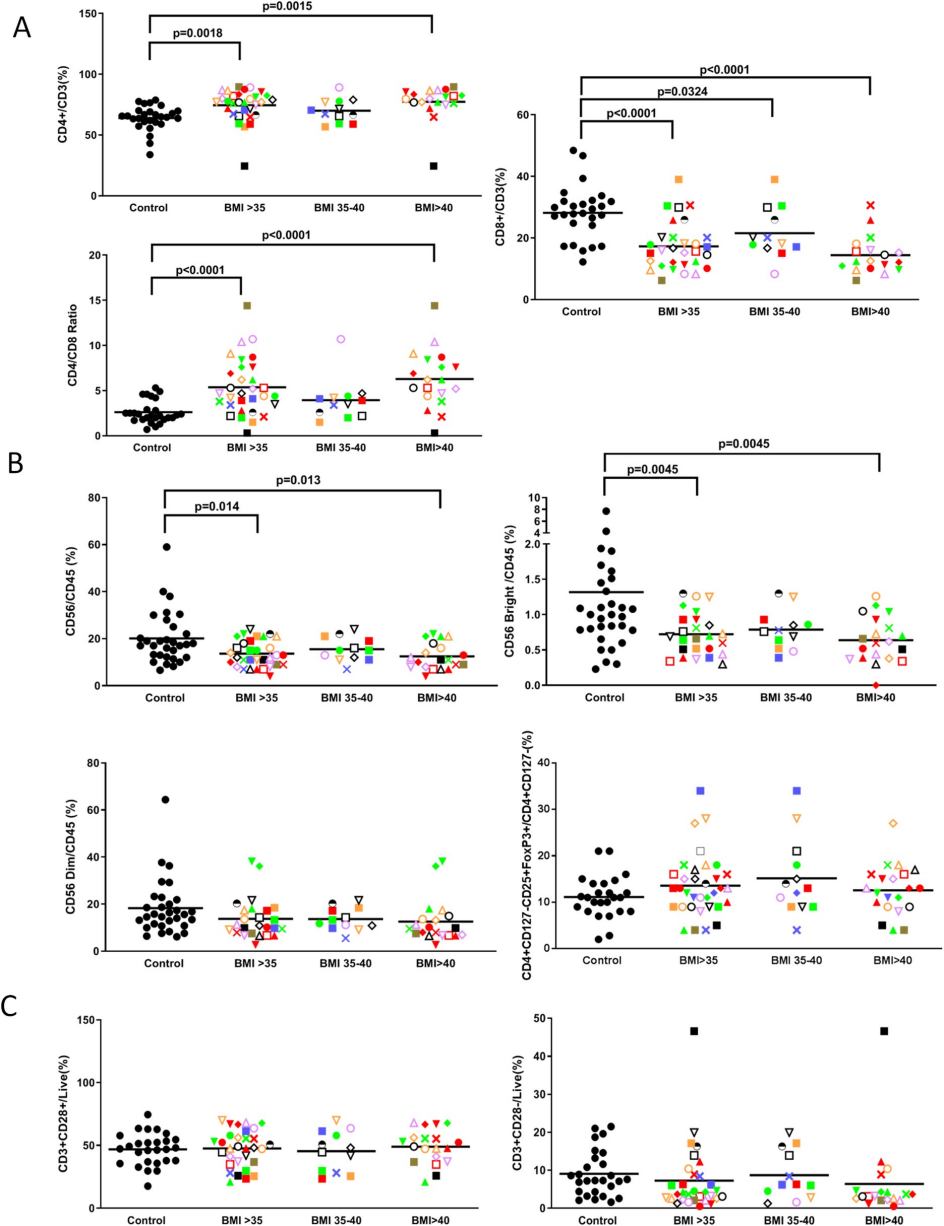
Obesity increases CD4+ T cells and Tregs and reduces NK and CD8+T cells. Volunteers with BMIs>35, each with a unique identifying symbol, were compared with controls for **(A)** CD4+ and CD8+ T cells and their CD4/CD8 ratio, **(B)** CD56/CD45, CD56 bright and dim NK cells and Tregs and **(C)** CD3**+** CD28+ and CD3+ CD28– T cells. Each volunteer with a BMI>35 is identified with a unique symbol. Results are expressed as the mean ± SEM.

Significant differences between the means of the cytokine/chemokine levels in the BMI>35 versus control BMI (18.5–24.9) group at time zero (i.e., to measure levels of CI), and in *E*. *coli* or HSV-1-stimulated blood samples were evaluated using unpaired t-tests when variances between the two groups were found to be equal, and Mann-Whitney tests for comparisons of groups with unequal variances. Exploratory analyses to identify significant differences between the BMI subgroups (BMI 35–40 and BMI>40) to the control group were subsequently identified using unpaired t-tests when variances between the two groups were found to be equal, and Mann-Whitney tests for comparisons of groups with unequal variances. The P values from the multiple comparisons between the different BMI groups and the control were corrected using the Benjamini and Hochberg’s false discovery rate of 5%. An adjusted P value <0.05 is considered statistically significant and reported in Figs 1–6. In addition, a correlation analysis between cytokines/chemokines, and BMI was performed using the statistic software Graphpad Prism 8.3.0 [15].

## Results

### Evaluation of plasma levels of the cytokines associated with chronic inflammation in the obese cohort

Forty two volunteers with BMIs>35 and 34 controls with normal BMIs (18.5–24.9) were recruited for this study and their characteristics are shown in Table 1. To measure levels of CI, plasma from freshly collected blood samples were analysed. Specifically, the levels of IL-1β, G-CSF, IL-10, IL-13, IL-6, IL-17, MIP1α, VEGF, IFNγ, IL-12p70, IFNα, IL-1RA, TNFα, IL-4 and IL-8 were measured using Luminex beads. As shown in Fig 1A, all of the volunteers with BMIs>35 were given a unique identifying symbol so that each individual could be tracked from one assay to another. As well, males were represented by different colored squares. Interestingly, only the level of IL-1 receptor antagonist (IL-1RA) was significantly (P<0.0015 different between volunteers with BMIs>35 and controls, with this cytokine being elevated in the obese cohort (Fig 1A). However, when the volunteers with BMIs>35 were subdivided into those with BMIs between 35–40 and those with BMIs>40 in exploratory analyses, a significant (P<0.0001) increase over the control group was only seen with the BMI>40 subgroup (Fig 1A). Given that IL-1RA competes with IL-1β for binding to the IL-1 receptor and prevents IL-1β-induced pro-inflammatory signalling we were interested in knowing if the increased production of IL-1RA in obese people was associated with high IL-1β levels. As shown in **Table 2**, there was indeed a significant (P<0.0001) correlation between IL-1β and IL-1RA levels (R value of 0.367). This was also demonstrated in **Fig 1A**, where 3 of the 4 people with the highest IL-1β levels (i.e., red diamond, inverted red triangle, red circle) also had the highest IL-1RA levels but the 4^th^ (green diamond) had a low IL-1RA level. As well, one person with a very high IL-1RA level (blue circle) had a very low IL-1β level, suggesting other factors may contribute to IL-1RA levels in these volunteers.

**Table 1:** Characteristics of volunteers with (n = 42) and without (n = 34) obesity.

Characteristic	Obese	Non-obese
Age (y)	56.3 ± 11.8	54.3 ± 11.8
Male %	26.2%	26.5%
Height (m)	1.67 ± 0.09	1.67 ± 0.09
Body mass (kg)	120.7 ± 22.3	64.4 ± 7.1
Body mass index (kg/m^2^)	43.4 ± 7.5	23.1 ± 1.4
Relative Fat Mass*	45.6 ± 7.6	37.9 ± 3.4
Type 2 diabetes	15	0
Hypertension	19	2

*Relative fat mass was calculated based on the formula RFM (men) = 64 –(20 x height/waist circumference); RFM (women) 76 –(20 x height/waist circumference) [16].

**Table 2:** Pearson correlation (R) values between parameters. Colored boxes indicate significant (P<0.05) correlations.

	CRP	BMI	Relative Fat Mass	PGE2	IL1ß	IL6	IL17	IL1RA	IL1RA (E coli)	IL1RA (HSV-1)	IL8 (HSV-1)	T-cell IFNγ	CD4/CD8	CD56/CD45	Granulocyte	RDW	
**CRP**																	
**BMI**	0.538																R =
**Relative Fat Mass**	0.458	0.682															>0.9
**PGE2**	0.291	0.139	0.063														0.8–0.9
**IL1ß**	0.022	0.055	0.159	-0.021													0.7–0.8
**IL6**	0.478	0.560	0.303	0.108	-0.057												0.6–0.7
**IL17**	0.028	0.387	0.002	0.282	-0.085	0.406											0.5–0.6
**IL1RA**	0.346	0.423	0.288	-0.238	0.367	0.237	-0.044										0.3–0.4
**IL1RA (E coli)**	0.299	0.532	0.085	0.037	0.023	0.436	0.433	0.192									0.2–0.3
**IL1RA (HSV-1)**	0.416	0.516	0.179	0.047	0.043	0.499	0.426	0.283	0.853								
**IL8 (HSV-1)**	-0.054	0.220	0.021	-0.306	0.299	0.100	0.237	0.334	0.271	0.375							
**T-cell IFNγ**	0.365	0.223	0.047	0.287	-0.138	0.271	-0.063	-0.099	-0.060	0.057	-0.272						
**CD4/CD8**	0.210	0.521	0.289	0.124	0.159	0.030	0.173	0.178	0.326	0.369	0.274	0.168					
**CD56/CD45**	-0.108	-0.401	-0.210	-0.163	-0.068	-0.167	-0.146	-0.187	-0.004	0.001	-0.116	-0.078	-0.198				
**Granulocyte**	0.657	0.512	0.142	0.240	-0.084	0.517	0.017	0.215	0.631	0.634	0.014	0.292	0.244	-0.274			
**RDW**	0.192	0.453	0.174	0.127	-0.080	0.194	0.190	0.001	0.065	0.044	0.142	0.174	0.321	0.014	0.182		

We also saw a significant (P<0.05) increase in IL-17, VEGF and IL-6 in the BMI>35 group but the levels of these 3 cytokines were just above their limits of detection in the Luminex assay. Thus, to corroborate these findings we retested these three cytokines using more sensitive MesoScale technology (see coefficient of variation (S1 Table) and lower limits of detection (S2 Table) for all the cytokines/chemokines tested in the two assays) and found, as shown in Fig 1B, that the levels of IL-17 and IL-6 were indeed significantly higher in the BMI>35 group, while there was a trend towards higher VEGF in the BMI>35 group than in controls. Since IL-17 has been shown to be increased in individuals with type 2 diabetes [17] we also compared the levels of IL-17 between the obese participants with and without type 2 diabetes and found that IL-17 was indeed significantly elevated in volunteers with type 2 diabetes (S1 Fig).

We were surprised to find that only a few of the 15 cytokines/chemokines tested in the Luminex/Mesoscale assays were significantly elevated with obesity. Those that were not are shown in S2 Fig. To obtain a more complete picture of the inflammatory status of our BMI>35 volunteers we also measured plasma CRP and PGE_2_ levels. We found that CRP, a well-established marker of CI [18], was significantly (P = 0.0001) elevated in the plasmas of the BMI>35 cohort and this difference was significant for both the BMI 35–40 (P = 0.0108) and BMI>40 (P<0.0001) subgroups in exploratory analyses (left panel, Fig 1C). PGE_2_ levels were also significantly elevated in the plasma of obese volunteers in both the BMI 35–40 (P = 0.0049) and BMI>40 (P = 0.0021) subgroups in exploratory analyses (right panel, Fig 1C).

### Elevation of innate immune responses to bacterial and viral challenges in the obese cohort

To challenge blood samples with agents that mimic *in vivo* infections, we employed intact *E*. *coli* and HSV-1, as in our earlier aging study [7], and incubated fresh, whole blood samples for 7 h to obtain levels of both early and late cytokines [7]. As shown in Fig 2, while whole blood samples stimulated with *E*. *coli* led to the production of high levels of IL-1β, IL-6 and IL-1RA in both control and BMI>35 samples (compare levels to those shown in Fig 1A & 1B), only IL-1RA was significantly (P<0.0001) higher in whole blood samples from the BMI>35 cohort, and this was specifically evident in the BMI>40 subgroup.

In response to HSV-1 stimulation, significantly (P<0.05) elevated levels of VEGF and IL-1RA, and a trend to an increased level of G-CSF and IL-8 were observed with the BMI>35 cohort (Fig 3), but only IL-1RA was significantly (P<0.05) elevated in the BMI>40 subgroup. The difference in the immune response to the HSV-1 and *E*. *coli* challenge is likely due to different Toll-like receptors being triggered on innate immune cells [19]. Of interest, Pearson correlation analyses revealed a statistically significant (P<0.0001) positive relationship between *E*. *coli* and HSV-1 stimulated IL-1RA levels (R = 0.853) but a weak correlation of these stimulated levels with endogenous IL-1RA levels (R = 0.192 and 0.283). In response to *E*. *coli* or HSV-1 stimulation, PGE_2_ levels were not significantly different between the normal and BMI>35 groups, whether obese volunteers were further subdivided in exploratory analyses into BMI 35–40 and BMI>40 or not (S3 Fig).

Whole fresh blood from 42 volunteers with BMIs>35 and 32 healthy people with BMIs between 18.5–24.9 were stimulated with HSV-1 for 7 h at 37°C and the conditioned plasma analyzed for cytokine/chemokine levels using Luminex beads. Each volunteer with a BMI>35 is identified with a unique symbol. Results are expressed as the mean ± SEM.

### Increase in activated T cell production of IFNγ in the obese cohort

To evaluate the effect of obesity on T cell activation, PBMCs were stimulated in 10% autologous plasma, in an attempt to simulate *in vivo* conditions, with anti-CD3 plus anti-CD28 for 4 days. As shown in Fig 4, T cells from volunteers with BMIs>35 secreted significantly (P = 0.0292) more IFNγ than the control group. This is consistent with a previous report showing that PMA + ionomycin stimulated PBMCs from an obese cohort stimulated higher IFNγ levels than from a control group [20].

### Increased RDW, monocytes and granulocytes in the obese cohort

Blood differential cell count analyses revealed that red blood cell distribution width (RDW), a measure of variation in red blood cell volume, was significantly (P<0.0001) higher in the BMI>35 group, and this difference was significant in exploratory analyses for both the BMI 35–40 (P<0.0001) and the BMI>40 (P = 0.0003) subgroups relative to the control group (left panel, Fig 5A). This is of interest since a high RDW has been associated with obese adolescents [21], metabolic syndrome, cardiovascular morbidity, all cause mortality, and ischemic stroke [22]. Consistent with this, Pearson correlation studies revealed a moderate correlation of RDW with BMI (R = 0.453) (Table 2). Blood differential cell count analyses also revealed that the mean corpuscular volume (MCV) of the red blood cells in the BMI>35 cohort tended to be lower. Typically, a high RDW coupled with a low MCV has been thought to be indicative of iron deficiency or microcytic anemia [23].

Flow cytometry was also carried out to look for differences in the proportions of various immune cells in volunteers with normal versus elevated BMIs. While there were no significant differences in lymphocytes (Fig 5B) or platelet numbers (**S4 Fig**), there was a significant (P = 0.0001) increase in total leukocytes in the BMI>35 group, specifically in granulocytes and monocytes (Fig 5B). This increase was significant (P<0.05) in exploratory analyses for both the BMI 35–40 and the BMI>40 subgroups. Pearson correlation studies revealed a significant (P<0.05) correlation between granulocyte numbers and CRP (R = 0.657), BMI (R = 0.512) and both *E*. *coli* (R = 0.631) and HSV-1 (R = 0.634) activated IL-1RA levels (Table 2).

### Increased CD4+ T cells and Tregs and reduced CD8+ T and NK cells in the obese cohort

The obese cohort also displayed a reduction in CD8+ and an increase in CD4+ T cells such that their CD4/CD8 ratio was more than double that of normal volunteers (Fig 6A). As well, the BMI>35 cohort had a significantly (P = 0.014) lower proportion of natural killer (NK) cells (i.e., for CD56 bright cells and a trend for CD56 dim cells) and a trend towards higher Tregs (CD4+CD127low/-CD25+FoxP3+ cells) (Fig 6B), suggesting a potentially poorer ability to eradicate cancer cells. Pearson correlation studies confirmed a moderate inverse correlation between NK cells and BMI (R = -0.401) (Table 2). Interestingly, there was no significant difference in the levels of CD3+CD28+T cells and CD3+CD28- T cells in the two cohorts (Fig 6C).

## Discussion

### Elevation of inflammatory markers in the obese cohort

Obesity is the number two risk factor for cancer mortality, after cigarette smoking [24]. Nonetheless, there are many people with obesity who do not succumb to cancer or other CI-related diseases [6] and it would be of great benefit to identify those at risk early on so that interventions can be initiated. With this in mind we carried out various assays to determine which would be the most informative at distinguishing those at risk. Looking at endogenous inflammatory markers, plasma levels of PGE_2_, CRP, IL-1RA, IL-17, and IL-6 were all significantly elevated in people with BMIs>35, which is consistent with previous reports suggesting obesity is pro-inflammatory and that this low grade CI is associated with an increased risk of cancer [5, 24–30]. On the other hand, there were a number of inflammatory cytokines/chemokines that we found were not affected by obesity, including IL-12, IFNα, IL-8 and MIP1α (S2 Fig). Moreover, we also observed that within the obese cohort there were people whose CRP, IL-1RA, IL-17, IL-6 and PGE_2_ levels fell within the normal range. Interestingly, an examination of specific obese individuals revealed that some with normal CRP levels also possessed normal levels of PGE_2_, IL-1RA, IL-17 and IL-6 (e.g., brown square, a man with a BMI>40). It thus appears that there is substantial person-to-person variation.

In terms of their roles in immune function, PGE_2_ has been shown to have both pro- [31] and anti-inflammatory [32] properties, complicating interpretation of its increase in those with BMIs>35. However, it is generally thought of as an inhibitor of both innate and adaptive immunity, in part by increasing myeloid derived suppressor cells (MDSCs) [32] and Tregs [33]. An increase might thus hinder the elimination of cancer cells but might be helpful in preventing autoimmune disorders. CRP, on the other hand, has been shown to be anti-bacterial by binding phosphocholine on the surface of bacteria [34] and damaged host cells, and activating the classical complement pathway to promote their phagocytosis [18]. As well, very recent studies suggest that CRP is not only capable of binding to leptin but may compete with it for binding to the leptin receptor, which may lead to leptin resistance [35] and, as a result, obesity. IL-1RA is a member of the IL-1 family that competes with the pro-inflammatory IL-1β for binding with the IL-1 receptor, thus antagonizing the effects of IL-1β-induced signalling [36]. In addition to being previously reported to be elevated in obesity [37], IL-1RA has been shown to be higher in human autoimmune and chronic inflammatory diseases [36]. Elevated IL-1RA may thus be part of a homeostatic attempt to return people to a less inflamed state. In keeping with this, we found a significant, albeit only weak, correlation between IL-1RA levels and IL-1β levels, suggesting that other factors may contribute to IL-1RA levels in our obese cohort. Gabay et al. for example, have proposed that obesity-associated factors such as leptin may also promote IL-1RA secretion from human monocytes [38]. It is thus possible that leptin, which is typically elevated in individuals with obesity, could augment the IL-1RA levels in these participants. It is interesting to note that even though IL-1RA is generally known as an anti-inflammatory cytokine, elevated IL-1RA levels may contribute to the development of insulin resistance [39], perhaps via a muscle-specific decrease in glucose uptake [40].

In addition, IL-6 was found to be elevated in the BMI>35 cohort. IL-6 is a prototypical pro-inflammatory cytokine associated with an increase in CI [36, 41] and implicated in mediating CI in people with obesity [41]. Moreover, it can increase VEGF levels and thus enhance neutrophil-mediated inflammatory responses by promoting transendothelial migration of neutrophils [42]. In obesity, the higher VEGF level may serve to increase adipose vasculature, thus preventing hypoxia in the growing fat mass and at the same time protect against insulin resistance [43, 44]. However, this same angiogenic property of VEGF can also enhance tumor growth, which may play a role in the increased cancer risk characteristic of people with BMIs>35 [45]. IL-17, like IL-6, is strongly associated with progression towards insulin resistance and type 2 diabetes in individuals with obesity [46]. It is also involved in the pathogenesis of autoimmune disorders [47] and plays a crucial role in promoting inflammation in adipose tissue of people with obesity [17]. As well, it has been shown to be increased in individuals with type 2 diabetes [17] which fits with our data showing that individuals with type 2 diabetes have significantly elevated levels of IL-17 compared to non-diabetic obese people (S1 Fig**).** Of note, there were no significant differences in weight between people with obesity with and without diabetes. Also of interest, the only significant difference between men and women with obesity that we found with our assays was an increase in IL-17 in men with obesity (S5 Fig). There was no significant difference in BMI between these two groups. This is relevant since men have a higher risk of liver cancer than women and current thinking is that liver cancer is triggered by IL-17 secreted by hypertrophic fat cells and promoting a cascade of hepatic inflammation [48–50]

### Increased innate and acquired immune responses in the obese cohort

Of the 15 cytokines/chemokines we examined, following 7 h stimulation with a bacterial or viral challenge, none were lower in the obese cohort, suggesting that innate immune responses were not compromised by obesity. In fact, blood cells of the BMI>35 cohort secreted significantly higher levels of VEGF, and non-significantly increased levels of G-CSF and IL-8 in response to HSV-1 stimulation. This is in contrast to our initial expectation that obese people would have a dampened immune response to bacterial and viral infection, since obesity is a risk factor for infections [2]. However, our finding is consistent with previous studies demonstrating an exaggerated inflammatory response in obese mice infected with bacteria or viruses [51–53]. Both the elevated basal and elevated HSV-1-stimulated levels of VEGF, the latter provoking a T_H_1 response like a nascent tumor cell, might enable a growing solid tumor, which typically becomes hypoxic, to survive by stimulating blood vessel infiltration into the tumor. As well, stimulating T cells with anti-CD3 plus anti-CD28 for 4 days suggested that T cells from those with a BMI>35 secreted IFNγ at significantly higher rates. This could be attributed, at least in part, to a significantly greater T-cell proliferation. While this, on the surface, might appear to contradict earlier studies showing that obesity increases the risk of infections and the outcome of infections once they are established [2], the more than 10-fold higher levels of IL-1RA than IL-1β observed in the plasma of the BMI>35 group, both endogenously and in response to microbial challenge, might counteract the ability of pro-inflammatory cytokines within the local infected milieu to destroy invaders. Whether this elevated innate and adaptive immune response leads to an increased risk of cancer remains to be determined. However, we hypothesize that this exaggerated inflammatory response in people with BMIs>35 results in collateral damage to normal cells, creating a favorable environment for initiation and/or promotion of cancer cell growth.

### Blood cells in the obese cohort

Our finding that the BMI>35 cohort has a significantly higher RDW and a non-significantly lower MCV is of interest since elevated IL-6 has been shown to promote liver production of the iron-regulatory peptide hormone hepcidin [54, 55]. An increase in hepcidin, in turn, is associated with reduced iron uptake from the intestine and increased iron trapping in liver cells and macrophages, thereby reducing iron availability for erythropoiesis [55, 56]. This ineffective erythropoiesis may in turn lead to reduced red cell size (i.e., decreased MCV) as well as a greater red cell size distribution (i.e., increased RDW), which is consistent with that found by Vuong et al with their obese participants [57]. As well, epidemiologic studies suggest that RDW is associated with increased levels of CRP and may be a predictor of chronic diseases and mortality in cardiovascular disease, cancer, and other diseases [58]. However, we only saw a weak correlation between RDW and CRP (R = 0.19187).

Our observation that granulocytes and monocytes are significantly elevated in our obese cohort is consistent with studies showing that adipocytes from abdominally obese individuals release alarmins (S100A8/A9) that trigger Toll-like receptor 4 on associated macrophages to secrete IL-1β that goes to the bone marrow to increase myelopoiesis. This, in turn, leads to increases in mature monocytes and granulocytes in people with obesity [59, 60]. Other obesity-associated factors such as leukotriene B4 have also been shown to stimulate neutrophil exit from the bone marrow [61].

We also found that, despite having a comparable total lymphocyte count, our obese cohort has reduced CD8+ and increased CD4+ T cells such that their CD4/CD8 ratio is markedly elevated compared to controls. This could lead to a lower ability to kill cancer cells. At the same time, there is a trend towards higher Tregs, which is consistent with a previous report of an increased level of CD4+CD25+FoxP3+ Tregs in the peripheral blood of the morbidly obese [62]. This contrasts with the finding that FoxP3+ Treg numbers are depleted in the visceral adipose tissue of the morbidly obese, while CD8 cytotoxic T cells are elevated [63, 64]. However, a systemic increase in immunosuppressive Tregs may be an adaptive response to counterbalance the local inflammation in the adipose tissue of obese individuals. Unfortunately, this increase in circulating Tregs might foster an environment favorable to cancer growth.

Our BMI>35 volunteers were also found to have a significantly lower proportion of CD56 bright and dim NK cells, two major NK subsets that are most notable for cytokine production and cytolytic activity, respectively [65]. This lower level of NK cells with obesity is in keeping with a previous study by D. O’Shea et al [66], but contrasts with Viel et al who reported an increased number of peripheral NK cells [67]. Of interest, a recent study suggests that obesity induces lipid accumulation in NK cells and that this blunts its anti-tumor response [68]. While we did not examine the effect of obesity on NK cell function, lower total NK cell levels coupled with an impaired NK function could lead to a potentially poorer ability to kill cancer cells.

Since there is increasing evidence that the gut microbiome is associated with the pathophysiology of obesity, we performed a preliminary study evaluating stool samples from 6 of our obese (BMI>40) and 6 controls to identify obesity-associated gut bacteria that may have value in predicting cancer risk. As expected, beta-diversity of the BMI>40 fecal microbiomes was significantly reduced (S6 Fig). As well, there was a dramatic increase in *E*. *coli* and a reduction in *Faecalibacterium prausnitzii* in the BMI>40 microbiome (S6 Fig). The increase in *E*. *coli* in the BMI>40 cohort was consistent with an earlier study showing elevated *E*. *coli* in obese school-aged Chinese children [69] and overweight pregnant women [70]. Since obese people tend to have a “leaky” gut, which allows lipopolysaccharide (LPS) to infiltrate into the blood and elevate CI [71], this elevation in *E*. *coli* might exacerbate this situation. The reduction in *Faecalibacterium prausnitzii* we observed with obesity is of interest since it is among the most abundant butyrate-producing bacterial species in the intestinal microbiota of healthy adults [72] and changes in its abundance have been linked to dysbiosis in several human disorders [72]. Whether changes in these specific bacteria may influence cancer risk needs to be validated in a larger study.

### Similarities and differences between obesity and aging

Since excess visceral fat has been reported to lead to an immunosenescence similar to that observed in elderly people [9, 10], we compared our current results with those from our earlier, normal aging study in which we used the same assays [7]. We found that, similar to normal aging, obesity is associated with significantly higher levels of CRP, PGE_2_, monocytes and granulocytes and significant reductions in NK cells. As well, both obesity and aging are associated with a marked increase in the CD4/CD8 T cell ratio and higher T cell IFNγ production in response to anti-CD3 plus anti-CD28 stimulation. However, unlike normal aging, obesity is associated with a higher RDW, perhaps because elevations in RDW are associated with metabolic syndrome rather than senescence [22], and also higher IL-1RA, IL-6, IL-17 and VEGF. As well, in response to *E*. *coli* or HSV-1 stimulation, PGE_2_ levels were not significantly different between our control and BMI>35 groups whereas PGE_2_ levels were dramatically reduced in our oldest cohort following *E*. *coli* stimulation and significantly elevated in response to HSV-1 [7]. Also, while there were no significant differences in platelet numbers between our normal and BMI>35 groups, they were reduced in our oldest age cohort [7]. In addition, in the current study we did not see the increase in IL-12 and decrease in G-CSF we observed with aging, highlighting important differences between obesity and normal senescence. As well, in contrast to our aging study where HSV-1-stimulated IFNα levels were markedly reduced with age [7], consistent with a reduced ability to generate an anti-viral response, they were similar in our control and BMI>35 groups. Perhaps this suggests that the mechanism by which old age and obesity increase the risk of cancer is distinct, with old age being due to an inability to mount an anti-viral immune response and obesity due to its creation of an immunosuppressive environment that is conducive for cancer cell growth.

### Individual variation of BMI>35 people

Having a unique symbol for each of the 42 obese volunteers has enabled us to track each person through the different assays and determine, to some degree, their relative risk. For example, one man with a BMI>40 (brown square) was found to be within the normal range in most of the assays we performed and thus may be at a low risk for CI and CI-induced diseases. In contrast, a BMI>40 woman with a high IL-1β level (red circle) had the highest CRP level, high IL-6 and high T cell stimulated IFNγ levels and markedly elevated granulocytes, putting her, perhaps, at very high risk. What is difficult to determine at this time is the relative weight to be given to the different markers and how many of these factors are elevated, besides IL-1RA, in an attempt to reduce inflammation. Carrying out these assays with a large cohort of people and following them over time should help to clarify this.

One of the limitations of our study is that we did not obtain information regarding dietary habits or physical activity from our volunteers. We are thus unable to determine if the obesity-related alterations in immune responses observed in our study are attributable solely to obesity or differences in lifestyle between the two groups. Also of note, while we did not carry out the study reported herein using one to one age-matched controls, we found that all the statistically significant differences we observed in our study were still statistically significant, except for monocyte levels, which lost significance when we compared one to one age-matched controls and BMI35-40 participants.

To conclude, we have identified several immune markers that are distinct in people with BMIs>35, including a number of pro-inflammatory cytokines that are elevated in the basal state as well as when challenged *ex-vivo* using bacterial or viral stimulation. Specifically, we found that basal levels of IL-1RA, IL-17, IL-6, CRP and PGE_2_ were elevated in our obese cohort, compared to our control group. When stimulated with *E*. *coli* and HSV-1, IL-1RA was consistently higher in the obese than the control group. We find it intriguing that some of the pro-inflammatory cytokines/chemokines, e.g. TNFα, IL-8 and IFN-γ that might be expected to be elevated were not higher in the obese group when compared to the lean cohort. The obese cohort evaluated in our study was also characterized by markedly higher granulocytes and monocytes and lower NK and CD8 T cell levels, which, when taken together, provide an immune cell profile that favors cancer development. To confirm the usefulness of these immune markers to predict cancer risk, a follow up study evaluating our participants’ health status over time is needed, and will be carried out.

## Supporting information

S1 Table Coefficient of Variation of the cytokines/chemokines using Luminex and Mesoscale. (PDF)

S2 Table Lower Limit of Detection of the cytokines/chemokines using Luminex and Mesoscale. (PDF)

S3 Table P values of correlation between parameters. Correlation coefficient is shown in Table 2.
(PDF)

S1 Fig IL-17 levels are significantly elevated in type 2 diabetic versus non-diabetic BMI>35 participants. (TIF)

S2 Fig Obesity did not affect several basal plasma cytokines/chemokines. (TIF)

S3 Fig PGE_2_ in response to *E*. *coli* or HSV-1 stimulation is not significantly different between controls and those with BMIs>35. (TIF)

S4 Fig Platelet numbers are not significantly different between controls and those with BMIs>35. (TIF)

S5 Fig IL-17 levels are significantly elevated in men with obesity compared to women with obesity. (TIF)

S6 Fig *E*. *coli* is significantly more abundant, while *Faecalibacterium prausnitzii* is reduced in BMI>40 participants. (TIF)
