# Supplementary material for: Exploratory examination of inflammation state, immune response and blood cell composition in a human obese cohort to identify potential markers predicting cancer risk
Source: PLoS One. 2020 Feb 6;15(2):e0228633. doi: 10.1371/journal.pone.0228633 (PMC7004330; doi:10.1371/journal.pone.0228633)
Supplement: S1 Table — (PDF) [file pone.0228633.s001.pdf]

S1 Table . Coefficient of Variation of Cytokines/Chemokines in Luminex and Mesoscale Analysis

Luminex Assay

IL-1β		G-CSF		IL-10		IL-13		IFN-γ		IL-12p70	
Conc. (pg/mL)	%CV	Conc. (pg/mL)	%CV	Conc. (pg/mL)	%CV	Conc. (pg/mL)	%CV	Conc. (pg/mL)	%CV	Conc. (pg/mL)	%CV
0											
6800	1.46	85100	2.92	13900	1			10960	0.07	8000	0.05
2267	3.49	28367	1.49	4633	0.64	4000	0.11	3653	5.58	2667	1.15
756	3.07	9456	2.86	1544	4.4	1333.33	4.76	1218	4.47	889	1.44
252	9.04	3152	1.58	515	3.3	444.44	2.17	406	4.18	296	4.77
84.0	6.4	1051	1.05	172	2.28	148.15	0.76	135	2.6	98.8	0.88
28.0	8	350	0.96	57.2	0.31	49.38	2.59	45.1	0	32.9	4.12
9.33	1.49	117	4.22	19.1	3.31	16.46	3.86	15.0	4.42	11.0	3.21
3.11	4.88	38.9	5.05	6.36	4.29	5.49	5.14	5.01	3.63	3.66	5.24

Luminex Assay

IFNα		IL-1RA		TNFα		IL-4		MIP-1α		IL-8	
Conc. (pg/mL)	%CV	Conc. (pg/mL)	%CV	Conc. (pg/mL)	%CV	Conc. (pg/mL)	%CV	Conc. (pg/mL)	%CV	Conc. (pg/mL)	%CV
8800	1.56	40150	0.33	7300	0.08	28400	1.14	18250	0.57	9500	3.99
2933	1.39	13383	0.72	2433	2.49	9467	2.53	6083	1.87	3167	11.36
978	2.71	4461	1.09	811	0.45	3156	0.53	2028	5.34	1056	0.25
326	5.59	1487	4.75	270	5.12	1052	0.66	676	7.97	352	1.17
109	2.39	496	8.25	90.1	2.77	351	3.38	225	1.13	117	0.47
36.2	7.29	165	2.66	30.0	4.99	117	3.9	75.1	7.11	39.1	4.93
12.1	7.44	55.1	6.33	10.0	3.29	39.0	4.31	25.0	3.21	13.0	6.83
4.02	0	18.4	1.23	3.34	0	13.0	0	8.34	0	4.34	4.68

Luminex Assay

IL-17		VEGF		IL-6	
Conc. (pg/mL)	%CV	Conc. (pg/mL)	%CV	Conc. (pg/mL)	%CV
19300	0.22	3400	1.4	5000	0.56
6433	2.77	1133	1.56	1667	18.56
2144	0.9	378	4.73	556	2.52
715	4.35	126	0.11	185	29.24
238	4.18	42.0	4.1	61.7	7.92
79.4	2.74	14.0	1.17	20.6	4.11
26.5	2.98	4.66	1.97	6.86	16.59
8.8	2.05	1.55	1.19	2.29	3.29

Mesoscale Assay

IL-17		VEGF		IL-6	
Conc. (pg/mL)	%CV	Conc. (pg/mL)	%CV	Conc. (pg/mL)	%CV
6480	4.3	1490	1.6	712	1.7
1620	2.7	373	2.1	178	2.1
405	2.9	93.1	2.9	44.5	3.1
101	3	23.3	3.9	11.1	2.2
25.3	3.1	5.82	2.7	2.78	1.8
6.33	2.9	1.46	4.5	0.695	2.3
1.58	6.5	0.364	7.9	0.174	3
0	15	0	11	0	4.3
