# Supplementary material for: Exploratory examination of inflammation state, immune response and blood cell composition in a human obese cohort to identify potential markers predicting cancer risk
Source: PLoS One. 2020 Feb 6;15(2):e0228633. doi: 10.1371/journal.pone.0228633 (PMC7004330; doi:10.1371/journal.pone.0228633)
Supplement: S2 Table — (PDF) [file pone.0228633.s002.pdf]

S2 Table. Lower limit of detection of the cytokines/chemokines analyzed using luminex and Mesoscale

		LLOD (pg/mL)
**Luminex**		
	IL-1β	3.11
	G-CSF	38.91
	IL-10	6.36
	IL-13	5.49
	IFN-γ	5.01
	IL-12p70	3.66
	IFNα	4.02
	IL-1RA	18.36
	TNFα	3.34
	IL-4	12.99
	MIP-1α	8.34
	IL-8	4.34
	IL-17	8.82
	VEGF	1.55
	IL-6	2.29
**Mesoscale**		
	IL-17	0.31
	VEGF	1.12
	IL-6	0.06
