# Supplementary material for: Exploratory examination of inflammation state, immune response and blood cell composition in a human obese cohort to identify potential markers predicting cancer risk
Source: PLoS One. 2020 Feb 6;15(2):e0228633. doi: 10.1371/journal.pone.0228633 (PMC7004330; doi:10.1371/journal.pone.0228633)
Supplement: S3 Table — Correlation coefficient is shown in Table 2. (PDF) [file pone.0228633.s003.pdf]

S3 Table. P values of correlation between parameters. Correlation coefficient is shown in Table 2.

	CRP	BMI	Relative Fat Mass	PGE2	IL1ß	IL6	IL17	IL1RA	IL1RA (E coli)	IL1RA (HSV-1)	IL8 (HSV-1)	T-cell IFNγ	CD4/CD8	CD56/CD45	Granulocyte	RDW
CRP																
BMI	0.000															
Relative Fat Mass	0.007	0.000														
PGE2	0.031	0.353	0.729													
IL1ß	0.853	0.655	0.377	0.884												
IL6	0.000	0.000	0.091	0.445	0.639											
IL17	0.811	0.001	0.992	0.037	0.467	0.000										
IL1RA	0.002	0.000	0.104	0.086	0.001	0.048	0.708									
IL1RA (E coli)	0.009	0.000	0.637	0.793	0.842	0.000	0.000	0.099								
IL1RA (HSV-1)	0.000	0.000	0.319	0.739	0.715	0.000	0.000	0.014	0.000							
IL8 (HSV-1)	0.661	0.088	0.918	0.039	0.013	0.437	0.051	0.005	0.025	0.002						
T-cell IFNγ	0.003	0.099	0.804	0.037	0.286	0.034	0.623	0.444	0.641	0.662	0.045					
CD4/CD8	0.119	0.000	0.121	0.407	0.252	0.832	0.202	0.198	0.016	0.006	0.063	0.230				
CD56/CD45	0.415	0.004	0.249	0.258	0.616	0.217	0.268	0.164	0.974	0.996	0.424	0.563	0.147			
Granulocyte	0.000	0.000	0.431	0.093	0.500	0.000	0.888	0.083	0.000	0.000	0.918	0.025	0.081	0.045		
RDW	0.117	0.000	0.333	0.381	0.521	0.121	0.121	0.994	0.602	0.729	0.282	0.188	0.020	0.921	0.135	

R=
>0.9
0.8-0.9
0.7-0.8
0.6-0.7
0.5-0.6
0.3-0.4
0.2-0.3
