# Supplementary figures and images for: Exploratory examination of inflammation state, immune response and blood cell composition in a human obese cohort to identify potential markers predicting cancer risk
Source: PLoS One. 2020 Feb 6;15(2):e0228633. doi: 10.1371/journal.pone.0228633 (PMC7004330; doi:10.1371/journal.pone.0228633)

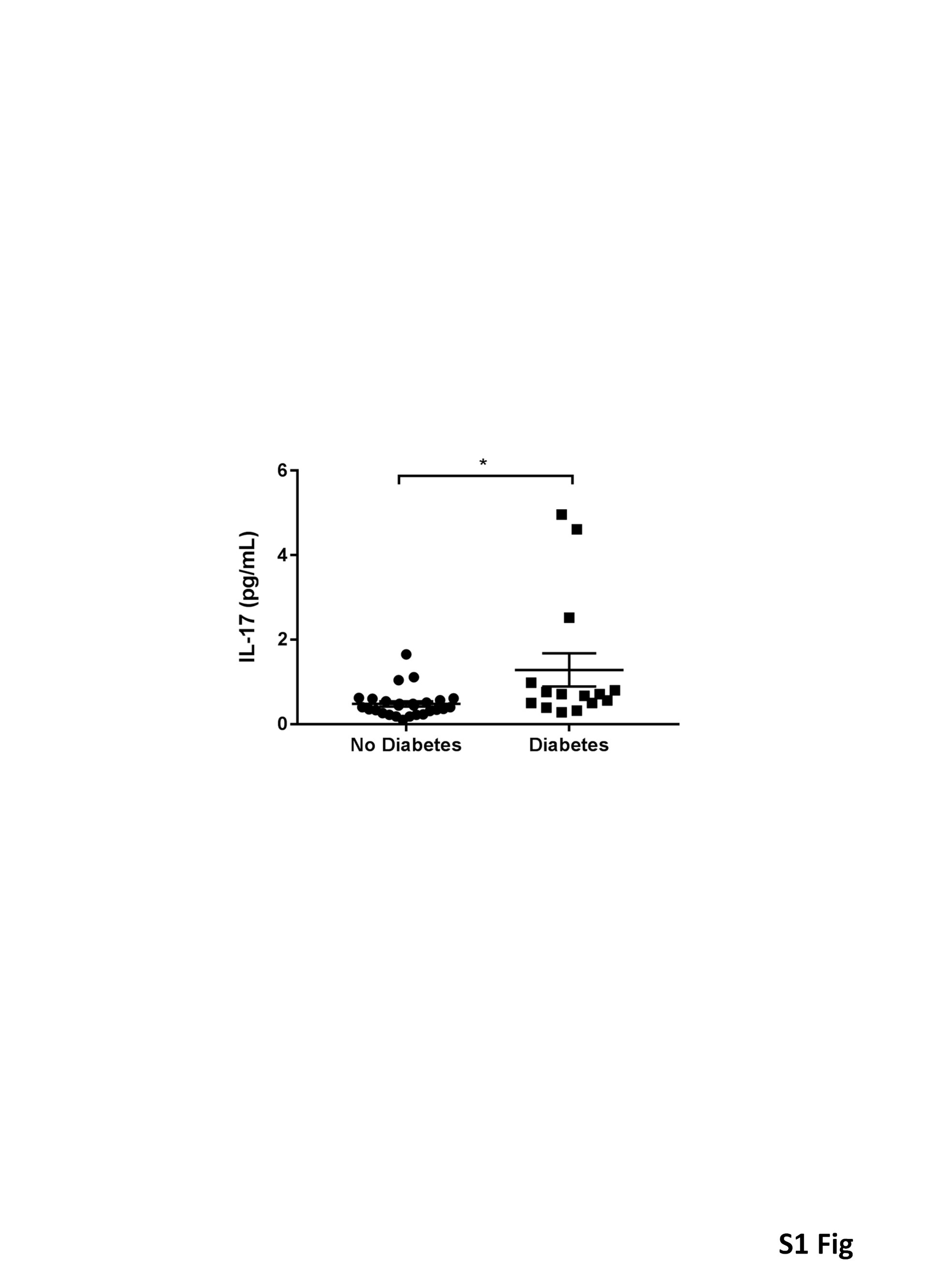

Supplement: S1 Fig — (TIF) [file pone.0228633.s004.tif]

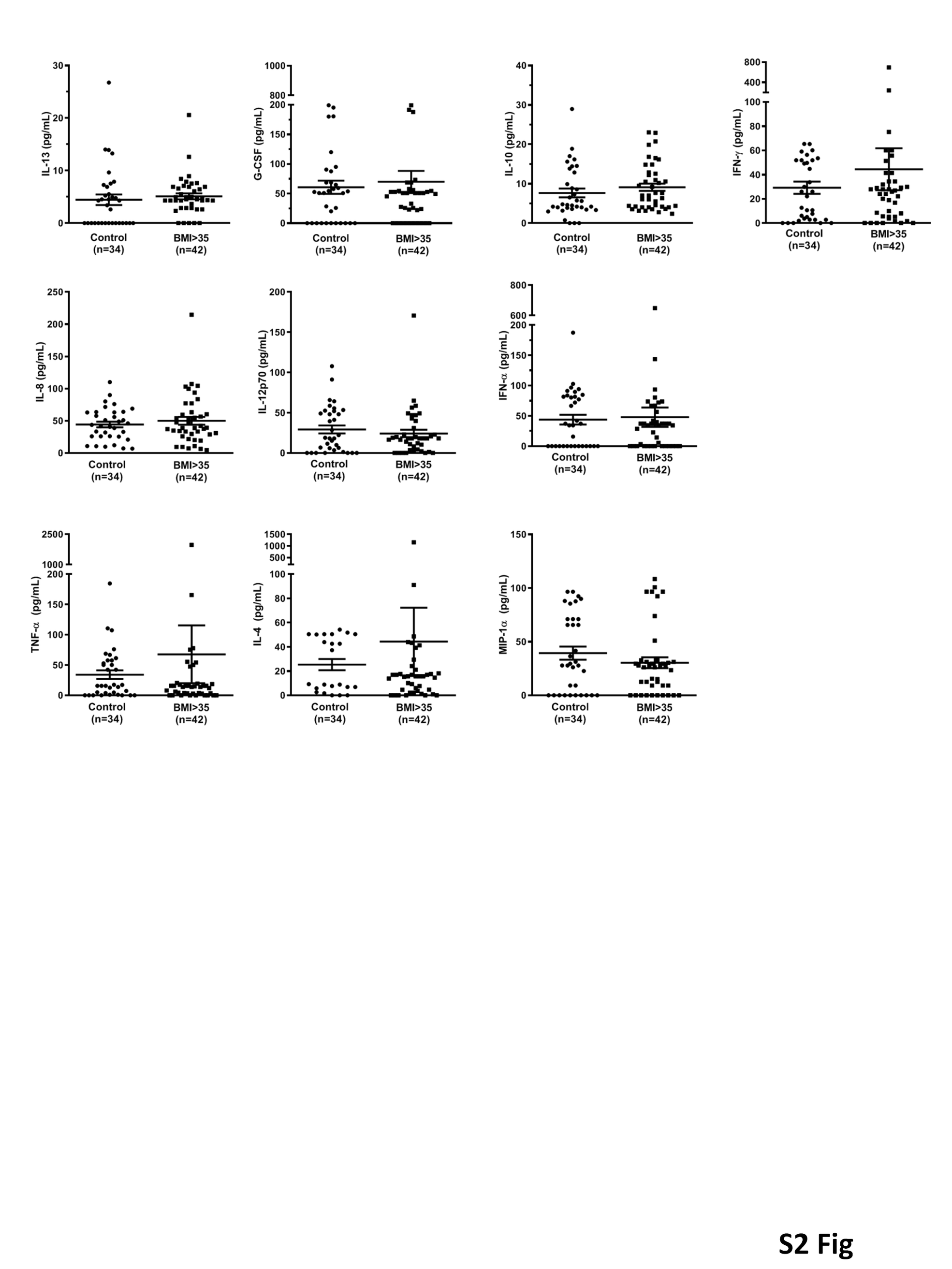

Supplement: S2 Fig — (TIF) [file pone.0228633.s005.tif]

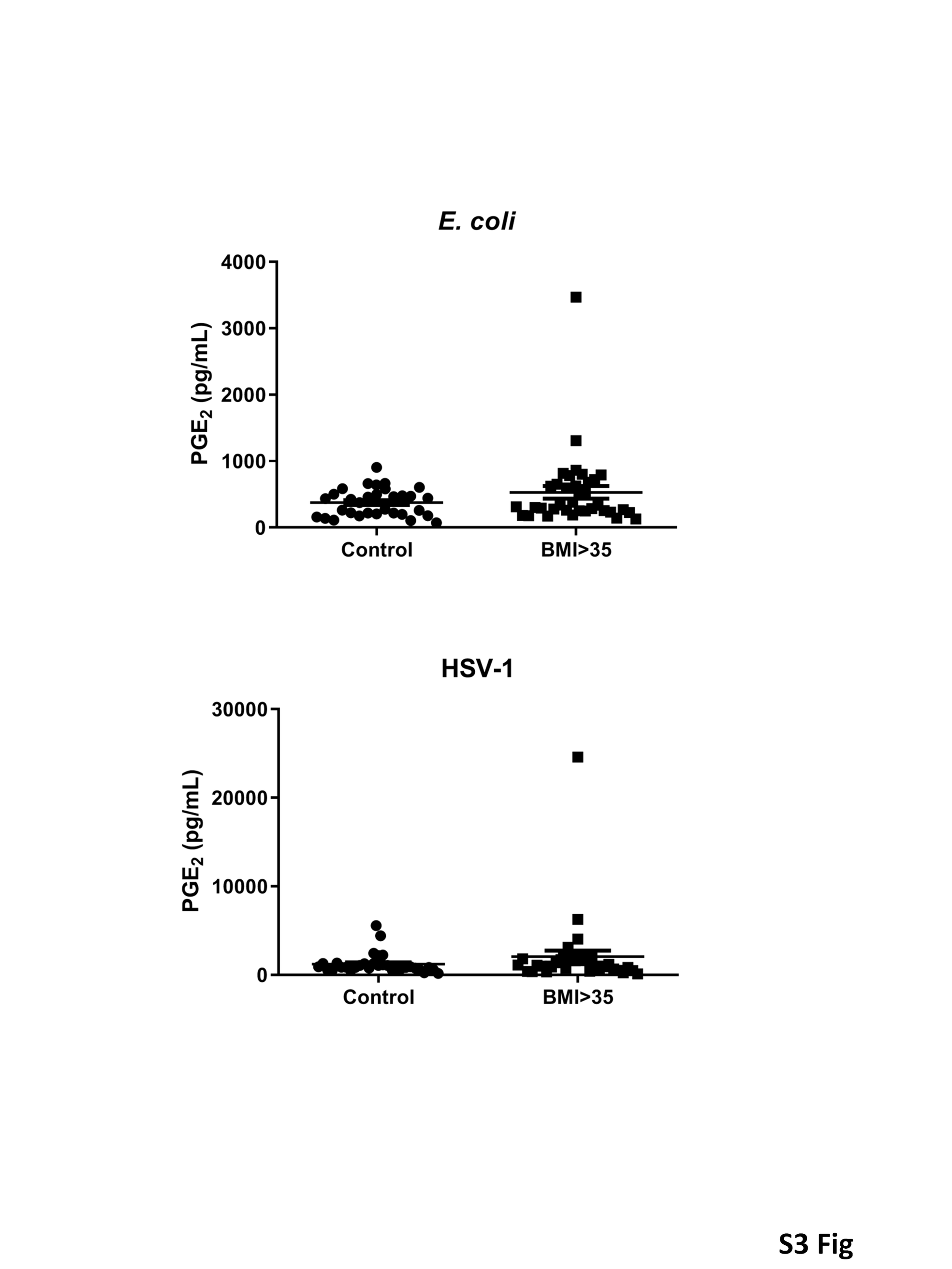

Supplement: S3 Fig — (TIF) [file pone.0228633.s006.tif]

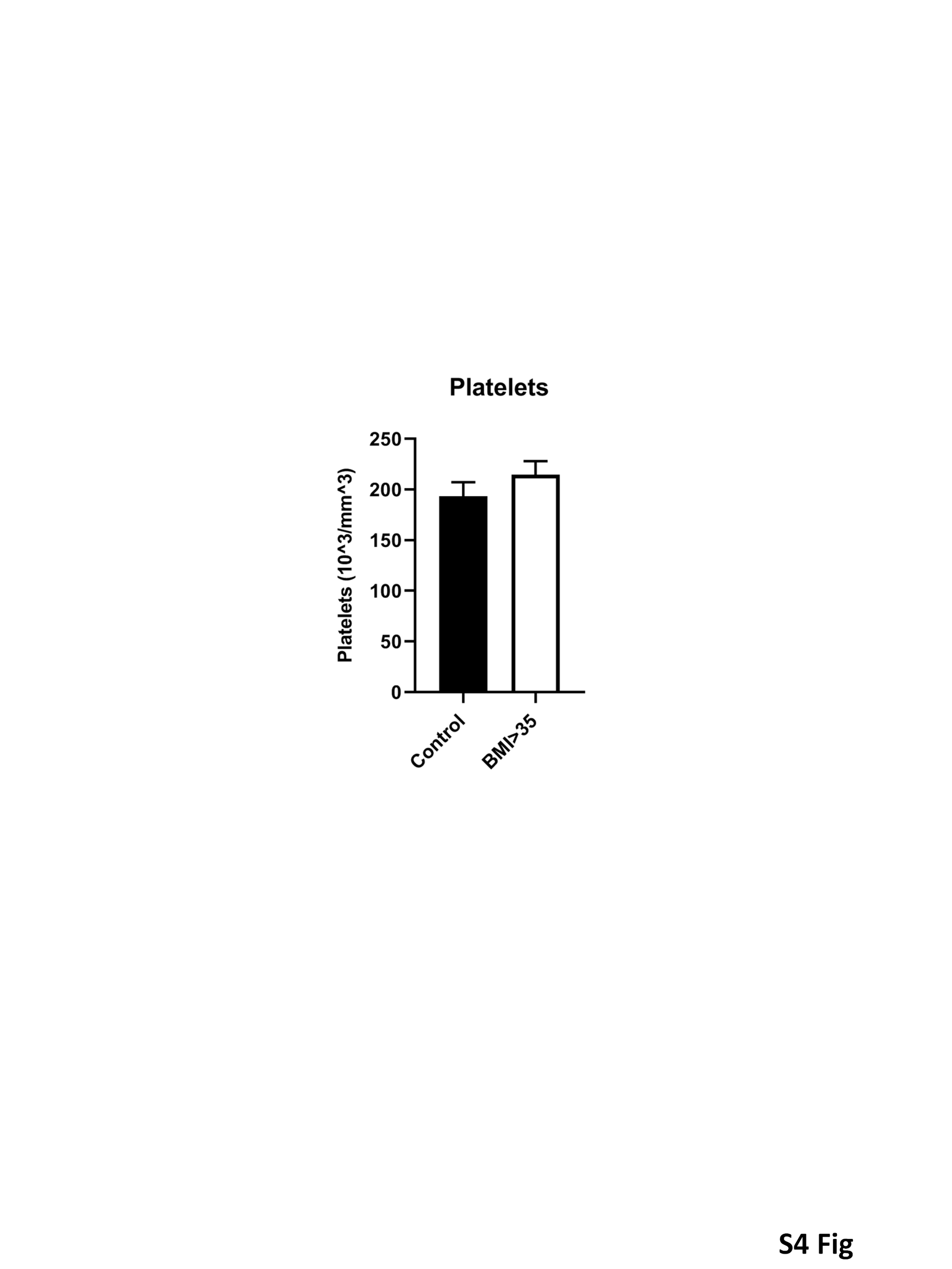

Supplement: S4 Fig — (TIF) [file pone.0228633.s007.tif]

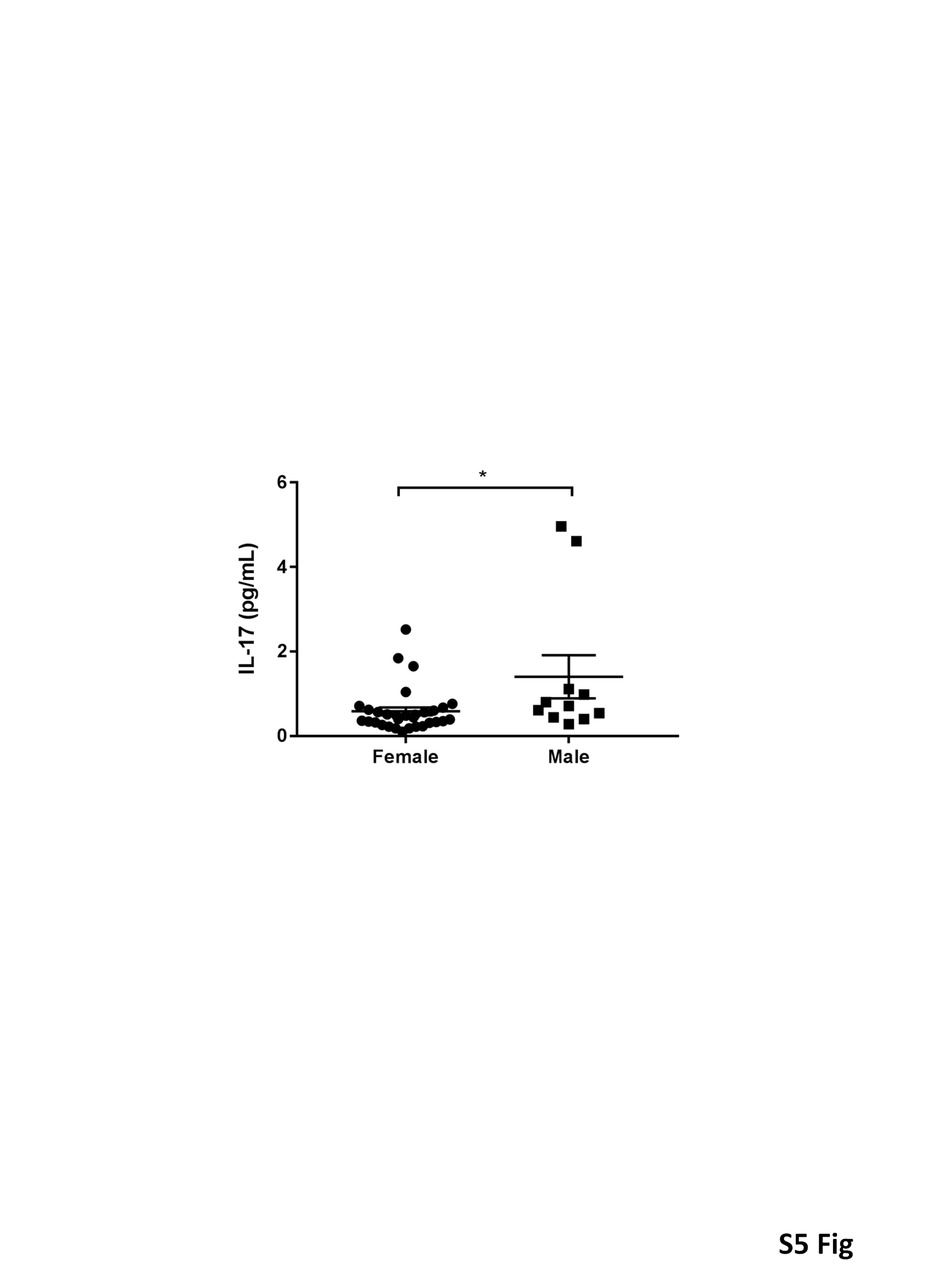

Supplement: S5 Fig — (TIF) [file pone.0228633.s008.tif]

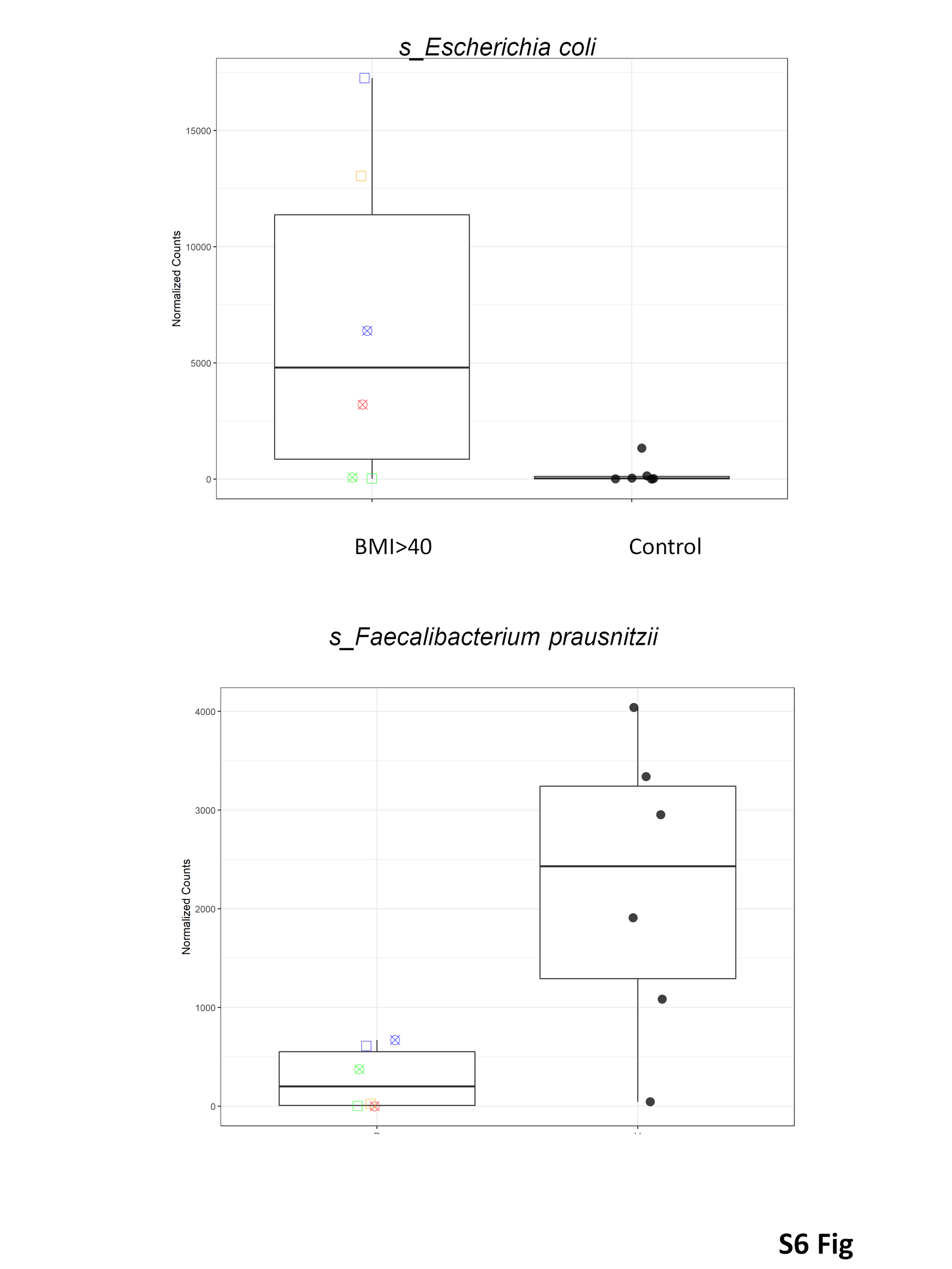

Supplement: S6 Fig — (TIF) [file pone.0228633.s009.tif]
